# Use of structural bone allograft in revision hip arthroplasty for massive acetabular defect: A systematic review and meta‐analysis

**DOI:** 10.1002/jeo2.70241

**Published:** 2025-05-03

**Authors:** Pietro Cimatti, Nicolandrea Del Piccolo, Alessandro Mazzotta, Benedetta Dallari, Enrico Pennello, Dante Dallari

**Affiliations:** ^1^ Reconstructive Orthopaedic Surgery Innovative Techniques ‐ Musculoskeletal Tissue Bank, IRCCS Istituto Ortopedico Rizzoli Bologna Italy; ^2^ Department of Medical and Surgical Sciences Bologna – University School of Medicine Alma Mater Studiorum Bologna Italy

**Keywords:** acetabular defect, hip, structural allograft

## Abstract

**Purpose:**

Managing substantial acetabular defects during revision total hip arthroplasty (rTHA) poses significant challenges, with a range of techniques available and ongoing discussions regarding their efficacy. This meta‐analysis aimed to assess the failure rates associated with Paprosky type III and American Academy of Orthopaedic Surgeons (AAOS) types III–IV acetabular defects treated with structural allografts in conjunction with cemented cups, cementless cups, or reinforcement devices.

**Methods:**

A systematic review was performed utilising PubMed/MEDLINE, EMBASE, and the Cochrane Database of Systematic Reviews to identify pertinent studies published from January 1980 to 1 April 2024. The search employed terms related to acetabular impaction bone grafting, rTHA, and associated techniques. The main outcome measure was the implant failure rate over an 8‐year period.

**Results:**

Twenty‐eight studies met the established inclusion criteria, covering three therapeutic approaches: (1) structural allograft with a cemented cup (four studies), (2) structural allograft with a cementless cup (10 studies), and (3) structural allograft with reinforcement devices (21 studies). The overall 8‐year implant failure rate was found to be 16% (95% CI, 11%–21%), with significant differences noted among the treatment modalities (*p* = 0.017). The failure rate was lowest for reinforcement devices (12%) and highest for cemented cups (30%). The predominant failure mechanism was aseptic loosening (68.9%), followed by infection (20.3%) and dislocation (10.8%). Rates of aseptic loosening were greater with cemented cups compared to cementless cups and reinforcement devices (19% vs. 13% and 6%, respectively; *p* = 0.023).

**Conclusions:**

Structural allografts combined with reinforcement devices yield favourable outcomes for managing large acetabular defects during revision THA, demonstrating significantly lower failure rates compared to other techniques. The addition of reinforcement devices substantially reduces the risk of implant failure.

**Level of Evidence:**

Level III.

**Abbreviations**
AAOS: American Academy of Orthopaedic Surgeon
rTHA: revision total hip arthroplasty
THA: total hip arthroplasty

## INTRODUCTION

Acetabular bone loss poses a considerable challenge in revision total hip arthroplasty (rTHA), especially with the increasing number of primary THAs performed in younger patients [51]. The need for acetabular revisions often arises from the loss of bone stock due to previous surgeries, prosthetic failures, and osteolysis stemming from wear particles of cement and polyethylene [24]. Severe structural deficiencies, such as Paprosky type 3A or 3B, along with conditions like pelvic discontinuity, add to the complexity of reconstruction efforts.

To tackle these challenges, various techniques have been developed, including impaction bone grafting, bulk bone grafting, artificial bone implantation, and the utilisation of jumbo cups without grafting. Devices like the Müller ring, Burch–Schneider reinforcement cage, and Kerboull reinforcement device are routinely utilised to support bone grafts [9, 19, 45]. Nonetheless, the reconstruction of type III acetabular defects, which account for up to 95% of acetabular revisions, remains difficult due to insufficient host bone available for implant support. Several methods, such as tantalum porous implants, reinforcement cages, rings and grafts, have been investigated for managing these defects [1]. Enhanced fixation approaches involving pelvic plates and metallic rings, like the Burch–Schneider cage, have led to improved outcomes. Although bone acetabular grafting can restore anatomy, leg length and bone stock for potential future revisions, its effectiveness—particularly concerning structural allografts—is still a topic of debate.

Structural cortical grafts, which possess approximately 70% of normal bone strength, are beneficial for bridging weight‐bearing defects but are also prone to complications due to revascularization. While revascularization is critical for host‐graft integration, it can weaken grafts due to remodelling. Typically, ingrowth is restricted to cancellous bone interfaces, though rare incidents of extensive revascularization have been reported over extended follow‐up periods [4, 5, 7, 14, 15, 22, 27, 29, 30, 34, 35, 43, 44, 45, 52].

Structural grafts can aid in restoring bone stock and reconstructing the acetabulum to its anatomical level; however, their efficacy remains contentious. While earlier studies indicated high failure rates for bulk allografts, more recent research has shown improved outcomes, particularly when graft load is kept below 50% of the cup support. Techniques such as the Kerboull acetabular reinforcement cross‐plate have shown potential in addressing complex defects by integrating both structural and morselized allografts [20, 25, 28, 33, 36, 39].

The antiprotrusio cage (APC), initially developed for protrusio acetabuli, has proven to be an effective solution for pelvic bone deficiencies. By dispersing joint forces and shielding grafts from mechanical overload, the APC bolsters structural support and promotes osseous integration [6, 8, 12, 17, 20, 26, 38, 40, 42, 52, 54, 55, 58]. However, allografts lacking support frequently fail due to resorption and loosening [13, 47, 49]. This study intends to systematically review and meta‐analyse the mid‐ to long‐term results of acetabular revisions utilising massive structural allografts. The primary goals include assessing implant survival and secondary outcomes such as aseptic loosening, infection, and dislocation. By consolidating the existing evidence, this research strives to enhance the understanding of bulk allograft‐based reconstructions and fill identified gaps in the literature.

## METHODS

### Search strategy

This systematic review and meta‐analysis was performed in accordance with the Preferred Reporting Items for Systematic Reviews and Meta‐Analyses (PRISMA) guidelines. The focus of this study was solely on the research question regarding structural bone grafting in acetabular revision, intentionally excluding clinical outcomes. A thorough search was carried out in PubMed/MEDLINE, EMBASE, and the Cochrane Database of Systematic Reviews for articles published from January 1980 to 1 April 2024. The search incorporated keywords pertinent to total hip arthroplasty, such as ‘acetabular bone defects’, ‘bone deficiency’, ‘structural bone allograft’, ‘bone graft’ and ‘revision total hip arthroplasty’. A systematic literature search in electronic databases was performed using combinations of the keywords mentioned above. Studies were screened independently by two review authors with a process to resolve differences. Only studies that evaluated the use of structural bone grafts for addressing substantial acetabular bone defects were included. The review was submitted for PROSPERO registration (ID 1008517) and data extraction was performed using extraction forms with specified outcomes with at least two review authors.

### Eligibility criteria

Two reviewers (P.C and A.M) independently evaluated the titles and abstracts of the identified studies. To be considered for inclusion, studies needed to report on outcomes of acetabular revisions utilising structural bone allografts for large acetabular defects defined as Paprosky types 3 A and 3B or AAOS types 3 and 4. The inclusion criteria (see Table 1) required the study to be a clinical trial discussing the use of structural allografts for acetabular defects, with no limitations based on language, publication date, or follow‐up length. Both prospective and retrospective studies were permitted, and the level of evidence was classified according to the Oxford Centre for Evidence‐Based Medicine guidelines. Studies involving fewer than ten patients, focusing solely on biomechanics, pertaining to femoral reconstruction, lacking radiological outcomes, or combining implant types without individual analysis were excluded (see Table 1).

**Table 1:** Selection criteria.

Inclusion criteria	Exclusion criteria
Treatment of massive acetabular bone defects	Treatment of minor acetabular bone defects
Use of structural bone allograft	Use of autograft or morselized allograft
Radiological outcomes	Studies with less than 10 patients

### Study selection and data extraction

**Figure 1:**
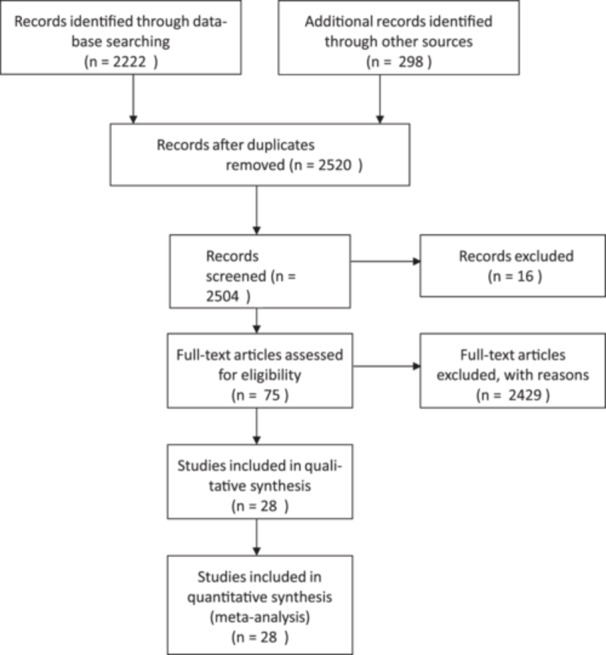
Prisma flow diagram.

The search and preliminary screening of abstracts were conducted by two independent reviewers, who resolved any discrepancies through discussion. Of the 2520 studies initially identified, 2504 remained after removing duplicates. A primary screening of abstracts narrowed this to 75 studies, which were then subjected to full‐text review. Ultimately, 47 studies were excluded due to the absence of structural allograft usage, leading to 28 studies included in the analysis (Figure 1). The quality of these studies was appraised using the Newcastle–Ottawa Quality Assessment Scale, with no minimum quality threshold established to promote inclusivity (Table 2).

**Table 2:** Quality assessment of the studies using Newcastle–Ottawa scale for cohort studies.

N. study	First author	Representativeness of the exposed cohort	Selection of the non exposed cohort	Ascertainment of exposure	Demonstration that outcome of interest was not present at start of study	Comparability of cohorts on the basis of the design or analysis	Assessment of outcome	Was follow‐up long enough for outcomes to occur	Adequacy of follow up of cohorts	Quality score
1	Schreurs et al. [48]	*	*	*	*	*	*	*	*	8
2	Stiehl et al. [51]	*	*	*	*	*	*	*	*	8
3	Schelfaut et al. [47]	*		*	*	*	*	*	*	7
4	Wedemeyer et al. [56]	*	*	*	*	*	*	*	*	8
5	Garbuz et al. [13]	*	*	*	*	*	*	*	*	8
6	Sporer et al. [50]	*		*	*	*	*	*	*	7
7	DeBoer [9]	*		*	*	*	*	*	*	8
8	Paprosky [36]	*	*	*	*	*	*	*	*	8
9	Brown et al. [3]	*		*	*	*	*	*	*	7
10	Traina et al. [53]	*	*	*	*	*	*	*	*	8
11	Kruger et al. [31]	*		*	*	*	*	*	*	7
12	Wegrzyn et al. [57]	*	*	*	*	*	*	*	*	8
13	Regis et al. [42]	*	*	*	*	*	*	*	*	8
14	Regis et al. [43]	*	*	*	*	*	*	*	*	8
15	Regis et al. [43]	*		*	*	*	*	*	*	7
16	Makita et al. [32]	*	*	*	*	*	*	*	*	8
17	Kerboull et al. [28]	*	*	*	*	*	*	*	*	8
18	Ilyas et al. [22]	*	*	*	*	*	*	*	*	8
19	Kawanabe et al. [27]	*	*	*	*	*	*	*	*	8
20	Schlegel et al. [46]	*		*	*	*	*	*	*	7
21	Goodman et al. [18]	*	*	*	*	*	*	*	*	8
22	Fujimoto et al. [11]	*	*	*	*	*	*	*	*	8
23	Gibon et al. [16]	*	*	*	*	*	*	*	*	8
24	Hsu et al. [21]	*	*	*	*	*	*	*	*	8
25	Piriou et al. [37]	*		*	*	*	*	*	*	7
26	Ishizu et al. [23]	*	*	*	*	*	*	*	*	8
27	Ebied et al. [10]	*	*	*	*	*	*	*	*	8

All the data were carefully extracted from all eligible studies independently by the two reviewers (P.C and A.M). The data extracted from the selected studies included various variables: study title, authors, year of publication, study design, sample size, surgical indications, type of bone allograft used, classification of acetabular defects, duration of follow‐up, rates of reoperation, and method of acetabular component fixation (cemented, uncemented press‐fit or with reinforcement devices). Additionally, outcomes such as radiographic loosening, revision rates and implant failures were noted. Any disagreement was resolved by discussion and consensus.

### Outcome measures

The primary outcome measure focused on the failure rates of acetabular implants, evaluated through the occurrence of re‐revisions. A forest plot was created to summarise pooled re‐revision rates across different studies. The secondary outcomes analysed included rates of aseptic loosening, infections, and implant dislocation.

### Subgroup analyses

Subgroup analyses were performed to explore the associations between re‐revision rates and various factors, including the type of acetabular defect (Paprosky or AAOS classification), fixation method (cemented, uncemented press‐fit or with reinforcement devices), and type of reinforcement used (cage, ring or mesh). Secondary outcomes, including aseptic loosening, infection rates and dislocations, were also evaluated.

### Quality assessment

Two investigators (P.C, A.M) independently evaluated the quality of the full texts with consensus discussion in case of discrepancies. Quality assessment was performed with the Newcastle–Ottawa Quality Assessment Scale. Studies that classified acetabular defects utilising Paprosky or AAOS systems were included, with large acetabular defects defined as AAOS types 3 and 4 or Paprosky types 3 A and 3B (Table 2).

### Risk of bias

Risk of bias was assessed using the Risk of Bias in Non‐randomised Studies of Interventions (ROBINS‐I) tool, which measures bias across seven domains. The seven items were listed as follows: (1) random sequence generation (selection bias), (2) allocation concealment (selection bias), (3) blinding of participants and personnel (performance bias), (4) blinding of outcome assessment (detection bias), (5) incomplete outcome data (attrition bias), (6) selective reporting (reporting bias), (7) other bias. Each item is evaluated as high, low, or unclear. We dealt with the disagreements by discussion, consulting with the third researcher if necessary (Table 3).

**Table 3:** Risk of bias assessment of the studies using ROBINS‐I tool.

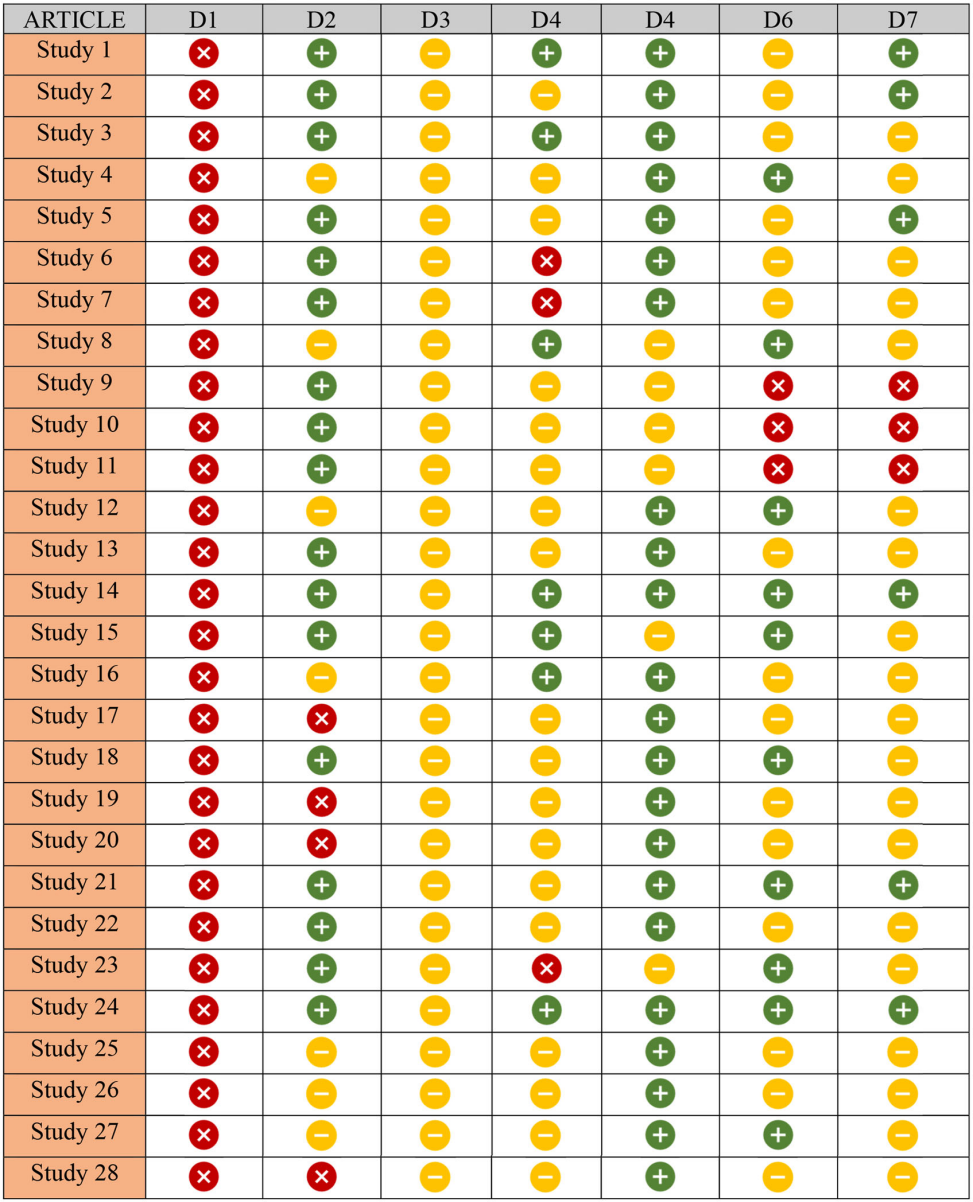

*Note*: Domains: D1: Random sequence generation (selection bias). D2: Allocation concealment (selection bias). D3: Blinding of participants and personnel (performance bias). D4: Blinding of outcome assessment (detection bias). D5: Incomplete outcome data (attrition bias). D6: Selective reporting (reporting bias). D7: Other bias.

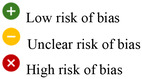

### Statistical analysis

The analysis considered the effects of individual studies, employing a random‐effects model for statistical analysis to compute the risk ratio and 95% confidence interval (95% CI). The null hypothesis (indicating a true effect size of 0) was rejected if the *p*‐value was below 0.05. To determine the contribution of sampling error versus the actual effect, heterogeneity was measured using Q statistics and degrees of freedom to calculate a *p*‐value that tests the null hypothesis concerning variations in effect size due to random sampling error. The null hypothesis was rejected if *p* was less than 0.05, indicating genuine variability in effects. Q statistics were also used to derive *I*², which presented the proportion of variation in effect sizes attributed to real differences rather than chance. An *I*² of 0 suggests that all variability in effect sizes is due to random sampling error. *I*² is expressed as a percentage of total variation across studies resulting from heterogeneity rather than random chance, and negative *I*² values are set to 0, keeping *I*² within a range of 0% to 100%. A value of 0% indicates no observed heterogeneity, while higher values suggest increasing levels of heterogeneity. Following established guidelines, an *I*² greater than 40% was regarded as indicating substantial heterogeneity.

## RESULTS

### Overall prosthesis failure rates

**Figure 2:**
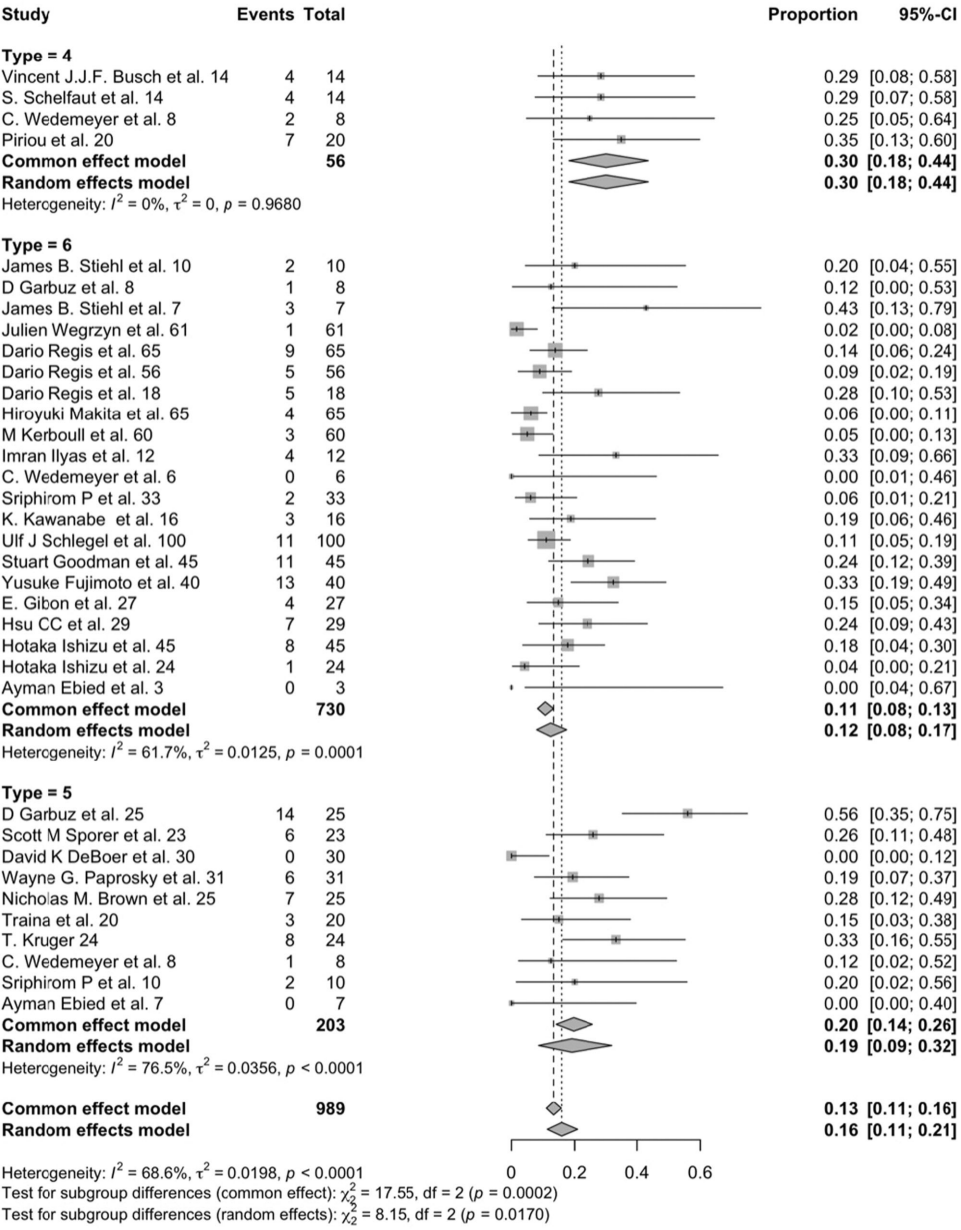
Forest plot for incidence of prosthesis failure in acetabular cemented cup (4), cementless cup (5) and antiprotrusio devices (6) (*p* = 0.017). CI, confidence interval.

A total of 28 studies, comprising 989 patients treated with structural allografts in combination with various surgical procedures (cemented cups, cementless cups, or antiprotrusio devices), were analysed using a random‐effects model. The overall incidence of prosthesis failure was 16% (95% CI: 11%–21%) over a mean follow‐up period of 8 years (Figure 2).

### Prosthesis failure by surgical procedure

- **Cemented cups with structural allografts**
  Four studies involving 56 patients used structural allografts in conjunction with cemented acetabular cups. The prosthesis failure rate in this group was 30% (95% CI: 18%–44%) (Figure 2).
- **Cementless cups with structural allografts**
  Ten studies, including 203 patients, employed cementless cups with structural allografts. The prosthesis failure rate in this group was 19% (95% CI: 9%–32%) (Figure 2).
- **Antiprotrusio devices with structural allografts**
  Structural allografts combined with antiprotrusio devices (e.g., cage, ring or mesh) were utilised in 21 studies involving 730 patients. The failure rate for this group was 12% (95% CI: 8%–17%) (Figure 2).

When comparing failure rates across surgical procedures involving structural allografts, there was a significant difference (*p* = 0.017). The lowest failure rate (12%) was observed with antiprotrusio devices, while the highest failure rate (30%) occurred with cemented cups (Figure 2).

**Figure 3:**
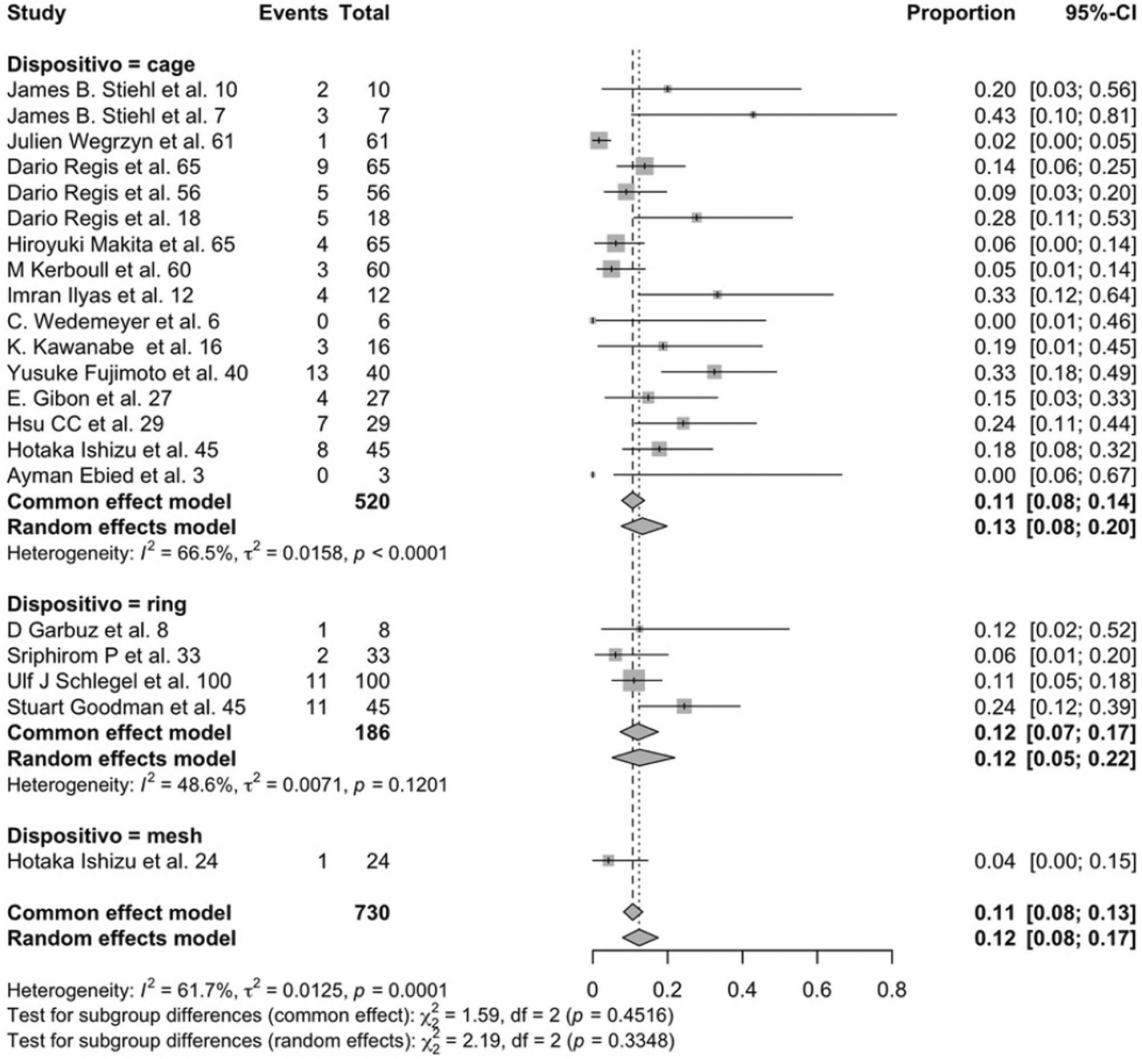
Forest plot for prosthesis failure rate in acetabular cup reinforced with cage, ring or mesh. CI, confidence interval.

A subgroup analysis comparing failure rates among antiprotrusio device types (cage, ring or mesh) showed no significant differences (*p* = 0.335) (Figure 3).

### Comparison of Paprosky Type 3A and 3B defects

**Figure 4:**
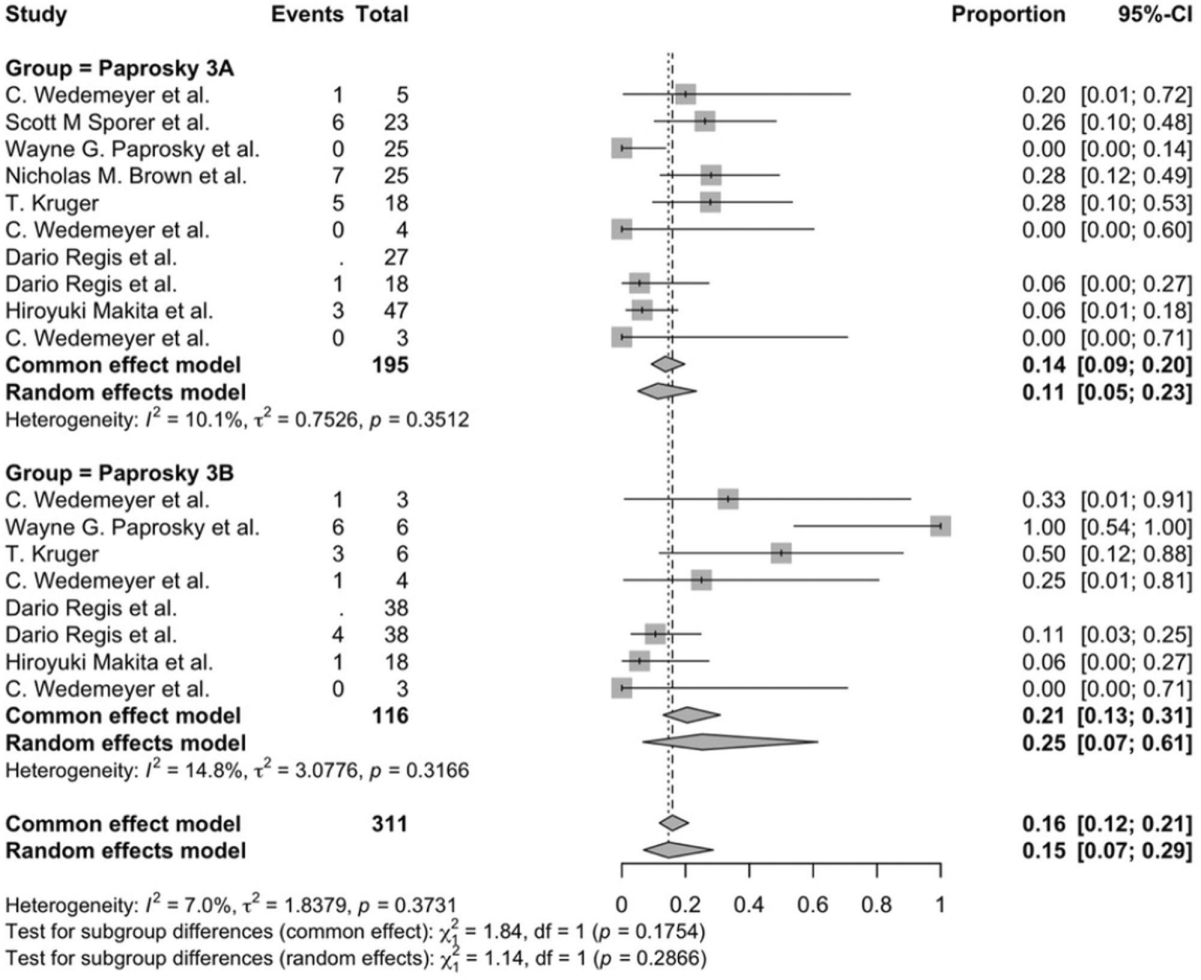
Mobilisation incidence: Paprosky 3A versus Paprosky 3B (*p* = 0.175). CI, confidence interval.

Data from 10 studies involving Paprosky type 3 A defects (195 patients) and eight studies involving Type 3B defects (116 patients) were analysed. The re‐revision rate for Type 3 A defects was 14% (95% CI: 9%–20%), while for Type 3B defects, it was 21% (95% CI: 13%–31%). The difference was not statistically significant (*p* = 0.175) (Figure 4).

### Surgical interventions by Paprosky defect type

The analysis of revision rates for different types of acetabular implants in managing Paprosky Type 3A and 3B defects yielded notable findings. For cemented cups, mobilisation rates were observed to be 20% (95% CI: 1%–72%) in patients with Type 3A defects, increasing to 33% (95% CI: 1%–91%) in those with Type 3B defects. However, this difference was not statistically significant (*p* = 0.676). In contrast, cementless cups demonstrated a significant variation in mobilisation rates, with 19% (95% CI: 12%–28%) for Type 3A defects, rising sharply to 62% (95% CI: 38%–82%) for Type 3B defects (*p* = 0.0007). Reinforcement devices exhibited relatively low mobilisation rates, with 6% (95% CI: 2%–15%) for Type 3A defects and 8% (95% CI: 4%–19%) for Type 3B defects, showing no statistically significant difference (*p* = 0.572).

### Leading causes of prosthesis failure

Among the recorded prosthesis failures (*n* = 161), the most common cause was aseptic loosening (68.9%, 103/161), followed by infection (20.3%, 36/161) and dislocation (10.8%, 15/161) (Table 4).

**Table 4:** Breakdown of prosthesis failure in different surgical procedures.

Failure Mode	Cemented cup	Cementless cup	Reinforcement devices	Overall
N. patients	56	203	730	989
Total failures	17 (30.36%)	97 (47.78%)	47 (6.44%)	161 (16.28%)
Aseptic loosening	11 (19.64%)	36 (17.73%)	56 (7.67%)	103 (10.41%)
Infection	3 (5.36%)	9 (4.43%)	24 (3.29%)	36 (3.64%)
Dislocation	3 (5.36%)	2 (0.99%)	10 (1.37%)	15 (1.52%)

### Infection and dislocation rates

**Figure 5:**
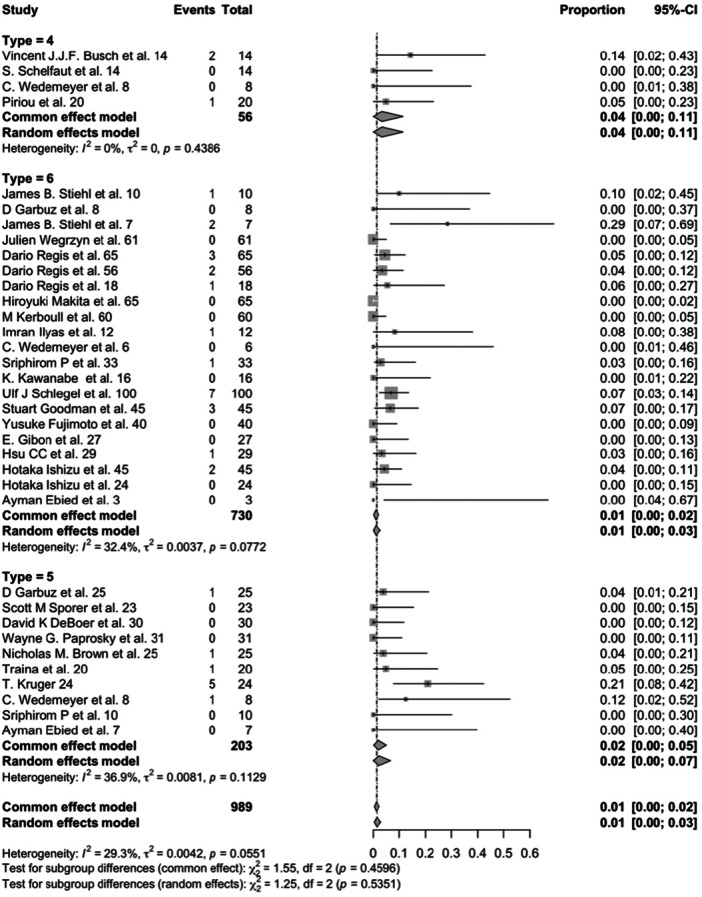
Forest plot for infection incidence in acetabular cemented cup (4), cementless cup (5) and antiprotrusio devices (6) (*p* = 0.535). CI, confidence interval.

**Figure 6:**
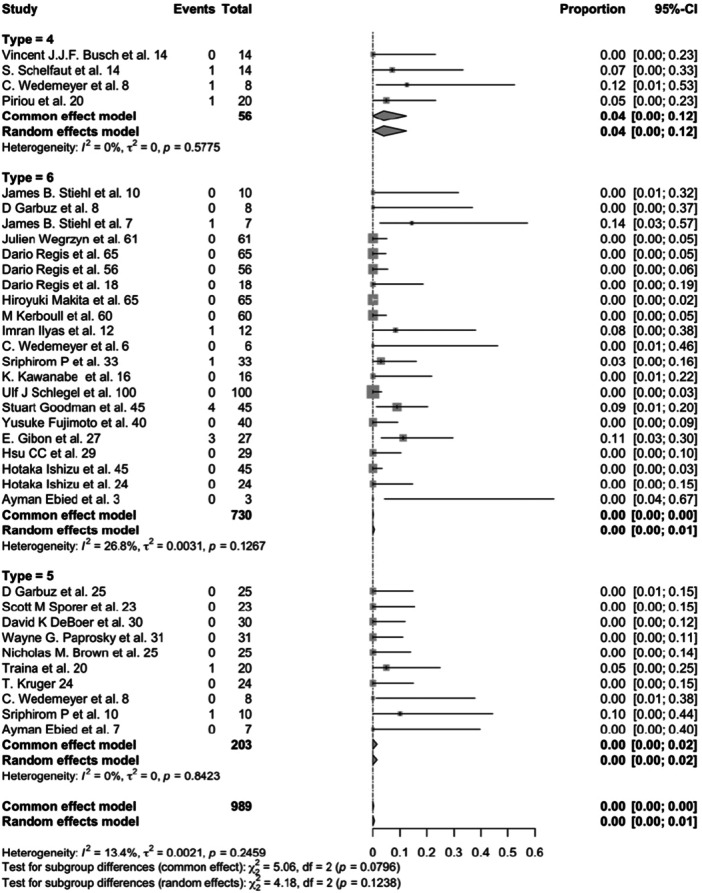
Forest plot for dislocation incidence in acetabular cemented cup (4), cementless cup (5) and antiprotrusio devices (6) (*p* = 0.124). CI, confidence interval.

Failure rates due to infection and dislocation did not differ significantly across interventions (*p* = 0.535 and *p* = 0.124, respectively) (Figures 5 and 6).

### Failure rates by surgical treatment

**Figure 7:**
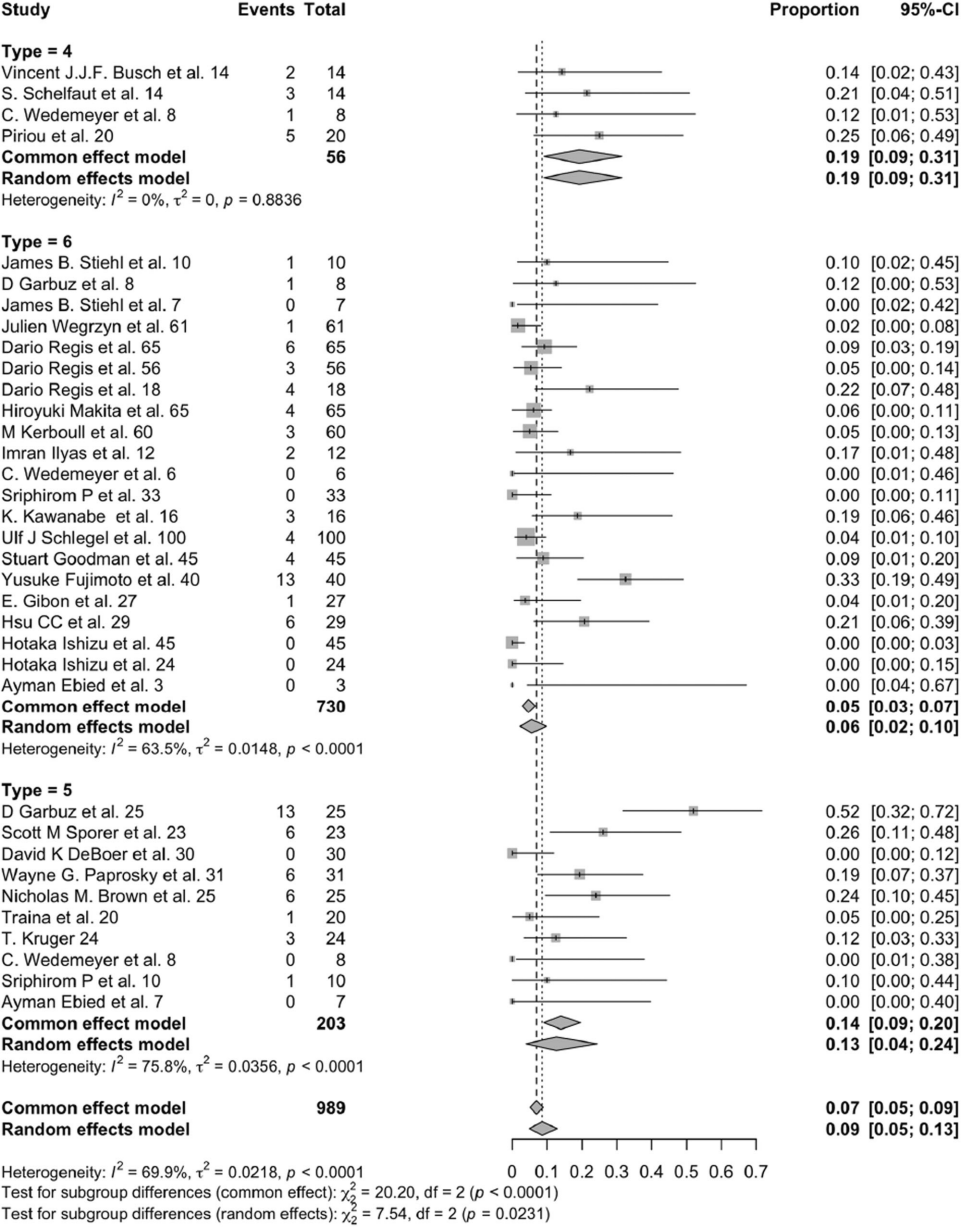
Forest plot for aseptic failure incidence in acetabular cemented cup (4), cementless cup (5) and antiprotrusio devices (6) (*p* = 0.023). CI, confidence interval.

The data from a total of 989 revision procedures were categorised based on the type of surgical treatment employed. Specifically, 56 revisions utilised cemented cups, of which 17 resulted in failure. In contrast, 203 revisions involved cementless cups, with 97 recorded failures. The largest group comprised 730 revisions employing reinforcement devices, which exhibited 47 failures. Notably, the rate of aseptic loosening was found to be significantly higher in cases involving cemented cups when compared to those utilising cementless cups or reinforcement devices (*p* = 0.023, Figure 7).

## DISCUSSION

The reconstruction of massive acetabular defects, particularly those classified as Paprosky Types IIIA and IIIB, remains a challenging endeavour in rTHA. This systematic review and meta‐analysis provides an updated synthesis of outcomes associated with structural bone allografts combined with different surgical strategies. Key findings from this study underscore the importance of surgical technique, implant type and reinforcement strategies in mitigating failure rates and improving long‐term outcomes.

### Comparative outcomes of reconstruction techniques

Structural bone allografts in combination with reinforcement devices have demonstrated the lowest failure rates (12%) over a mean follow‐up period of 8 years. In particular, Wegrzyn et al. [57] reported no mechanical failure of the Kerboull cross‐plate and complete osseointegration of the allograft in 60 of the 61 acetabular reconstructions (98%). Conversely, Stiehl et al. [51] documented an overall revision rate of 47%, with failures due to infection in two type IVB patients and one type IVC patient, as well as implant loosening in four cases (two type III posterior and two type IVB). Additionally, one type IVB patient experienced recurrent implant dislocation. The four cases of loosening were revised to a cemented all‐polyethylene component, with all implants initially placed on <50% host bone. Notably, no re‐revisions occurred in these cases [51].

Following Wegrzyn et al.'s [57] findings, Regis et al. [41] reported that nine out of 65 reconstructive APCs required re‐revision during the follow‐up period, with an additional four cases showing radiographic loosening. The cumulative survival rate was 80.0% (95% CI, 72.6%–88.1%) after 18.9 years, further supporting the long‐term efficacy of these reconstructive techniques [41].

The efficacy of antiprotrusio devices, such as cages and rings, in enhancing stability and reducing aseptic loosening is well‐supported. These devices likely provide mechanical advantages by distributing loads more evenly across the graft and host bone, thereby mitigating the risk of graft resorption and mechanical failure.

In contrast, cemented cups exhibited a significantly higher failure rate of 30%, with aseptic loosening being the predominant mode of failure. This aligns with previous studies highlighting the mechanical disadvantages of cemented fixation in compromised bone stock. Schelfaut et al. [47] reported a 67.1% survival rate at five years, using revision for any reason as the endpoint. On the other hand, Schreurs et al. [48] in their Kaplan–Meier analysis, found a 20‐year survival rate of 80% (95% CI, 67%–94%) with acetabular revision for any reason as the endpoint, and 91% (95% CI, 80%–100%) when considering revision due to aseptic loosening. This suggests that acetabular reconstruction with impaction bone grafting and a cemented polyethylene cup is a durable and reliable technique, particularly in young patients with acetabular bone‐stock defects [48]. Similarly, Piriou et al. [37] defining success as the survival of the bulk allograft without radiologic evidence of progressive graft collapse and a PMA score better than 15, reported a 65% success rate, while 35% of cases required revision due to graft resorption or infection.

Although cementless cups performed better than cemented ones (failure rate 19%), their outcomes remained inferior to those achieved with reinforcement devices, emphasising the limitations of press‐fit fixation in cases of severe bone loss. Traina et al. [53] examined 23 patients with pelvic bone stock deficiency involving major columns who underwent revision surgery with a cementless press‐fit cup and a structural bone graft. Among 20 patients followed for at least six years (average 7.6, range 6–11 years), three cups required revision: one for aseptic loosening, one for septic loosening, and one for recurrent dislocation. The Kaplan–Meier cumulative probability of survival without revision for loosening at 11 years was 84.4% [53]. Brown et al. [3] reported a lower survival rate in a cohort of 14 cup revisions using structural periacetabular allografts, with a Kaplan–Meier survivorship of 67.1% at 42 months. In agreement with Traina et al. [53], Sporer et al. [50] found a 10‐year survival rate of 78% (95% CI, 74%–82%) when considering re‐revision due to aseptic loosening, and 74% (95% CI, 70%–78%) when radiographic signs of loosening were included as endpoints.

A key factor influencing aseptic loosening in cementless arthroplasty is biological fixation. Apostu et al. [2] emphasised the importance of optimising osseointegration and minimising micromotion at the bone‐implant interface to prevent aseptic loosening. Their review highlighted strategies such as surface modifications, advanced biomaterials, and pharmacological approaches to enhance fixation and longevity in cementless arthroplasty [2].

Overall, the current evidence underscores the advantages of reinforcement devices in achieving superior long‐term outcomes, while cementless fixation strategies continue to evolve to address the challenges associated with severe bone loss.

### Factors contributing to failure

Aseptic loosening was identified as the leading cause of failure (68.9%), followed by infection (20.3%) and dislocation (10.8%). The higher aseptic loosening rates observed with cemented cups may stem from the challenges of achieving durable fixation in deficient bone stock, particularly in cases of pelvic discontinuity or extensive segmental defects. Infection rates, while consistent across groups, remain a critical concern given their potential to compromise both graft integration and implant stability. Dislocations, though less common, were more frequent in cases involving Paprosky type IIIB defects, likely reflecting the inherent challenges of restoring proper hip biomechanics in these cases.

### Subgroup analysis and clinical implications

Interestingly, no significant differences in failure rates were observed between Paprosky type IIIA and IIIB defects treated with structural allografts. However, when stratified by implant type, Paprosky type IIIB defects demonstrated a significantly higher rate of mobilisation when treated with cementless cups compared to type IIIA defects (62% vs. 19%, *p* = 0.0007). This finding underscores the need for more robust fixation strategies, particularly in type IIIB defects, where pelvic discontinuity and severe bone loss exacerbate the challenges of achieving durable fixation.

Reinforcement devices demonstrated superior outcomes across both defect types, suggesting their utility as a preferred option for managing massive acetabular defects. Subgroup analysis revealed no significant differences in performance among different types of reinforcement devices (cage, ring or mesh), suggesting that device selection can be guided by surgeon preference and specific anatomical considerations.

## CONCLUSIONS

This meta‐analysis highlights the pivotal role of reinforcement devices in achieving durable outcomes in rTHA for massive acetabular defects. Structural bone allografts combined with these devices result in significantly lower failure rates compared to cemented or cementless cups alone. Aseptic loosening remains the predominant mode of failure, emphasising the need for strategies that enhance initial fixation and promote graft integration.

For cases involving Paprosky type IIIB defects, reinforcement devices offer distinct advantages by addressing the biomechanical challenges associated with severe bone loss and pelvic discontinuity. Surgeons should prioritise these devices in such scenarios to optimise patient outcomes.

Future research should focus on refining graft integration techniques, exploring the potential of emerging biomaterials, and conducting prospective randomised studies to further delineate the comparative effectiveness of available reconstruction strategies. In the interim, this study provides a robust evidence base to guide surgical decision‐making in the management of massive acetabular defects.

## AUTHOR CONTRIBUTIONS

The author(s) read and approved the final manuscript.

## CONFLICT OF INTEREST STATEMENT

The authors declare no conflicts of interest.

## ETHICS STATEMENT

Not available due to literature based.

## Data Availability

The data sets used and analysed during the current study are available from the corresponding author on reasonable request.
